# Ensemble Learning for Breast Cancer Lesion Classification: A Pilot Validation Using Correlated Spectroscopic Imaging and Diffusion-Weighted Imaging

**DOI:** 10.3390/metabo13070835

**Published:** 2023-07-11

**Authors:** Ajin Joy, Marlene Lin, Melissa Joines, Andres Saucedo, Stephanie Lee-Felker, Jennifer Baker, Aichi Chien, Uzay Emir, Paul M. Macey, M. Albert Thomas

**Affiliations:** 1 Radiological Sciences, University of California Los Angeles, Los Angeles, CA 90095, USA; ajinjoy@mednet.ucla.edu (A.J.); timshel@g.ucla.edu (M.L.); mjoines@mednet.ucla.edu (M.J.); asaucedo@mednet.ucla.edu (A.S.); stlee@mednet.ucla.edu (S.L.-F.); achien@mednet.ucla.edu (A.C.); 2 Physics and Biology in Medicine-Inter-Departmental Graduate Program, University of California Los Angeles, Los Angeles, CA 90095, USA; 3 Surgery, University of California Los Angeles, Los Angeles, CA 90095, USA; jlbaker@mednet.ucla.edu; 4 School of Health Sciences, College of Health and Human Sciences, Purdue University, West Lafayette, IN 47907, USA; uemir@purdue.edu; 5 School of Nursing, University of California Los Angeles, Los Angeles, CA 90095, USA; pmacey@sonnet.ucla.edu; 6 BioEngineering, University of California Los Angeles, Los Angeles, CA 90095, USA

**Keywords:** correlated spectroscopic imaging, diffusion weighted imaging, machine learning, breast cancer, choline, myo-inositol, glycine, water, lipids

## Abstract

The main objective of this work was to evaluate the application of individual and ensemble machine learning models to classify malignant and benign breast masses using features from two-dimensional (2D) correlated spectroscopy spectra extracted from five-dimensional echo-planar correlated spectroscopic imaging (5D EP-COSI) and diffusion-weighted imaging (DWI). Twenty-four different metabolite and lipid ratios with respect to diagonal fat peaks (1.4 ppm, 5.4 ppm) from 2D spectra, and water and fat peaks (4.7 ppm, 1.4 ppm) from one-dimensional non-water-suppressed (NWS) spectra were used as the features. Additionally, water fraction, fat fraction and water-to-fat ratios from NWS spectra and apparent diffusion coefficients (ADC) from DWI were included. The nine most important features were identified using recursive feature elimination, sequential forward selection and correlation analysis. XGBoost (AUC: 93.0%, Accuracy: 85.7%, F1-score: 88.9%, Precision: 88.2%, Sensitivity: 90.4%, Specificity: 84.6%) and GradientBoost (AUC: 94.3%, Accuracy: 89.3%, F1-score: 90.7%, Precision: 87.9%, Sensitivity: 94.2%, Specificity: 83.4%) were the best-performing models. Conventional biomarkers like choline, myo-Inositol, and glycine were statistically significant predictors. Key features contributing to the classification were ADC, 2D diagonal peaks at 0.9 ppm, 2.1 ppm, 3.5 ppm, and 5.4 ppm, cross peaks between 1.4 and 0.9 ppm, 4.3 and 4.1 ppm, 2.3 and 1.6 ppm, and the triglyceryl–fat cross peak. The results highlight the contribution of the 2D spectral peaks to the model, and they demonstrate the potential of 5D EP-COSI for early breast cancer detection.

## 1. Introduction

Breast cancer is one of the most prevalent cancers in females and one of the leading causes of cancer death worldwide [1,2]. Early detection and accurate characterization of breast malignancies are crucial factors in breast cancer management and positive treatment outcomes [3,4,5,6,7,8,9,10,11,12]. Differentiation of benign from malignant breast lesions can aid clinicians in determining appropriate therapeutic plans. While histopathological examination of breast tissues extracted by biopsy is often required to confirm a suspicious lesion, a mammogram continues to be the gold standard for the detection of breast cancer, but this approach has a high false positive rate [13]. Multi-parametric MRI (mp-MRI), which includes dynamic contrast-enhanced MRI (DCE-MRI), T_2_-weighted MRI and diffusion-weighted imaging (DWI) may allow differentiation between benign and malignant breast lesions that present highly overlapping enhancement patterns. However, despite the potential to eliminate unnecessary biopsies and follow-up examinations of benign tumors, mp-MRI-based breast tumor differentiation still has increased false positive findings.

Cell density, organization, membrane integrity and cellular metabolism of breast tissues undergo changes in the presence of cancer. Magnetic resonance spectroscopic imaging (MRSI) is capable of detecting the changes in concentrations of various metabolites and lipids in the tissue that are altered due to cancer-related changes in cellular metabolism [14,15,16,17,18,19,20,21,22,23]. High cell density and altered tissue structure due to cancer also lead to restricted motion of water molecules in the tissue, which can be measured by the apparent diffusion coefficient (ADC) on DWI [12,24,25,26,27,28,29,30,31]. DCE-MRI, one of the most sensitive diagnostic techniques, highlights the areas of increased blood flow and blood volume in the breast tissues due to cancer with the help of a contrast agent [5,6,7,12,32,33,34,35].

Even though the sensitivity of mp-MRI methods can be affected by various factors like tumor size and aggressiveness, these methods are often reported to have relatively high sensitivity (in the range of 88–100% for DCE-MRI, 85–95% for DWI and 80% for MRSI) [9,12,36,37,38,39]. Reported specificity, on the other hand, is relatively low (69–74% for DCE-MRI, 75–82% for DWI and 74% for MRSI), restricting the capability for classification of benign and malignant lesions [37,38,39,40]. While single-voxel spectroscopy has a reported 64–82% sensitivity and 85–91% specificity [41], the multi-voxel technique of MRSI can cover a larger area of the breast with a relatively higher spatial resolution. Advanced MRSI techniques like five-dimensional (5D) echo-planar correlated spectroscopic imaging (EP-COSI) can record two-dimensional (2D) correlated spectroscopy (COSY) from multiple regions in three-dimensional (3D) space [42]. Achieving high specificity is also challenging in MRSI due to overlapping patterns of the measures between benign and malignant lesions.

One option to potentially improve the specificity while retaining the benefits of the non-invasive nature of these imaging modalities is to use machine learning (ML) models to identify subtle or complex differences in the multi-model data that differentiate benign and malignant lesions [43,44]. Development and validation of machine learning models have seen impressive growth in the last decade due to their high accuracy and flexibility in handling a wide range of data types and features [45]. While individual machine learning models may perform well, a meta-approach that combines individual models named ensemble learning could generate even more generalizable models that can reduce individual base learner’s variance or bias [46]. In particular, advanced ensemble models like the gradient-boosted tree-based algorithm that combines multiple weak learners (decision trees) are shown to be capable of detecting key features of the multi-modal, multi-parametric imaging information for applications such as tissue/cancer grade classification [47,48,49,50].

Multiple studies have recently shown that the features extracted from DCE-MRI and DWI of breast tissues used in ML models are capable of predicting tumor grades and classifying benign and malignant breast lesions [48,49,50]. However, metabolite and lipid information from MRSI data has not been used in this context so far. Therefore, a major goal of this work was to evaluate the application of different machine learning models, including ensemble learning techniques, for the classification of benign and malignant breast lesions based on the 5D EP-COSI data along with the corresponding ADC information from DWI data.

## 2. Materials and Methods

### 2.1. Subjects and Data Acquisition

The dataset consisted of 5D EP-COSI and DWI data from twenty-three subjects with malignant breast masses (mean age 53 [range: 33–71] years and seventeen benign breast masses (mean age 37 [range: 19–60] years). All scans were acquired on a Siemens 3T Skyra scanner (Siemens Healthineer, Erlangen, Germany). Consent was obtained from all volunteers included in the study according to the on-site institutional review board guidelines. The 5D EP-COSI data was acquired using FOV = 160 × 160 × 120 mm^3^, matrix size = 16 × 16 × 8, TR/TE = 1500/35 ms, 64 t_1_ points and 512 t_2_ points with a spectral width of 1250 Hz and 1190 Hz along F_1_ and F_2_, respectively. A non-water-suppressed (NWS) 1D MRSI scan with one t_1_ point was acquired for eddy current phase correction and for combining signals from multiple receiver coils [51]. The data was non-uniformly undersampled (NUS) along two spatial k_y_-k_z_ and the spectral t_1_ dimensions with a total acceleration factor of 8, and was reconstructed using a Group Sparsity (GS)-based compressed sensing technique [52,53].

The DWI acquisition protocol included the following: Two-dimensional spin-echo echo-planar imaging (EPI) sequence (TR/TE of 3800/93 ms; data matrix, 192 × 192; signal average, 3; slice thickness, 3 mm; and distance factor, 20%) in the axial plane. Diffusion sensitizing gradients (DSG) in three orthogonal directions with b values of 50 and 800 s/mm^2^ were applied. The ADC maps were created automatically by the in-line scanner software using the trace-weighted images with b values of 50 and 800 s/mm^2^.

### 2.2. Pre-Processing

Tumor-containing slices in the DWI were selected and the boundaries of the lesion were marked by a radiologist. ADC values were then extracted from this delineated region of interest (ROI). The MRSI data were interpolated by a factor of 2 and the slices containing the tumor were identified similarly to DWI. Spectroscopic voxels within the delineated region were extracted and the metabolite and lipid ratios were quantified in these voxels as described in [42]. All variables were standardized with z-score normalization (zero mean and unit standard deviation) and voxels containing outlier measurements were removed. For the variables that followed a normal distribution, outliers were identified as three standard deviations away from the mean. For other variables, previously reported ranges of metabolite and lipid ratios were used as a guideline for outliers [42].

### 2.3. Feature Extraction

Ten to twelve voxels from multiple slices were selected for each MRSI dataset, resulting in 241 malignant voxels and 195 benign voxels after removing outliers. The Apparent Diffusion Coefficient value was calculated for each dataset and assigned to voxels under the respective dataset.

A total of 99 features were available for the study. These were derived from both DWI and MRSI data as follows:
DWI: 1 feature (ADC).1D MRSI: 3 features, includes water fraction (water/(water + fat)), fat fraction (fat/(water + fat)) and water-to-fat ratio (water/fat).2D MRSI: 95 features which are the ratios of 24 metabolite and lipid peaks with respect to 4 different reference peaks. Reference peaks include methylene fat, olefinic fat and water at 1.4 ppm, 5.4 ppm and 4.7 ppm from the 1D spectrum, and the methylene fat diagonal peak at 1.4 ppm from 2D spectrum. These constitute to 96 features, out of which the ratio of 2D Methylene Fat diagonal peak (FAT14) with itself is excluded resulting in 95 features.

**Figure 1:**
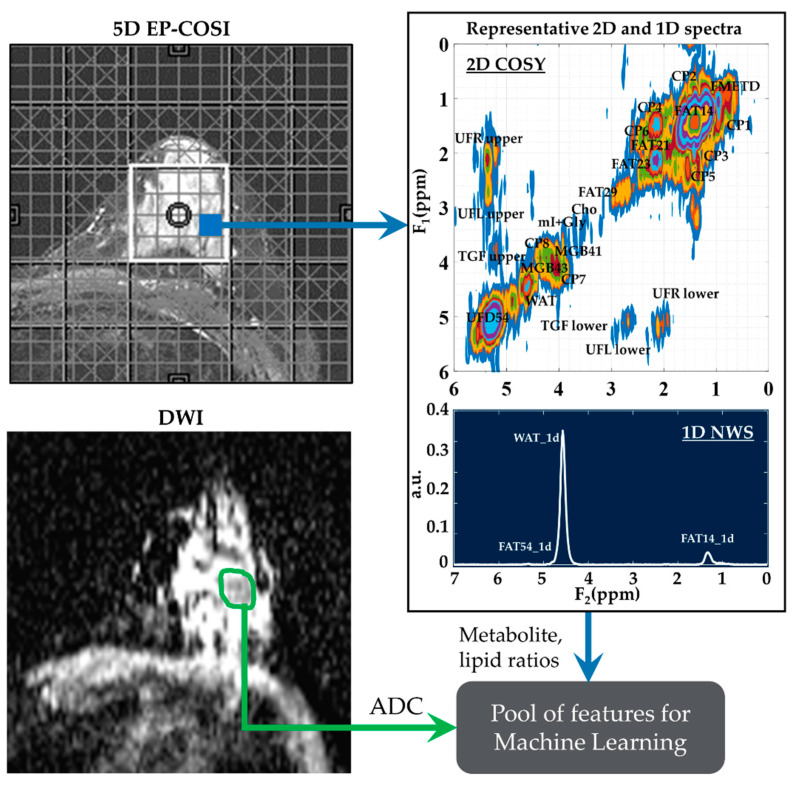
A representative 2D COSY spectrum and ADC map. Localizer image for 5D EP-COSI acquisition is shown on the top left panel. White box represents the placement of the volume of interest. An extracted COSY spectrum and 1D NWS spectrum are shown on the right side. Bottom-left panel shows the corresponding ADC map for the same subject with the region of lesion marked in green. These metabolite, lipid ratios and ADC values were inputted into the feature pool, which was then narrowed down using statistical tests, recursive feature elimination and sequential forward selection.

These features were then narrowed down using statistical tests and feature selection algorithms. The full list of metabolites and lipids including choline (Cho), myo-Inositol + glycine (mI + Gly), unsaturated fatty acid and triglyceryl fat cross-peaks identified in the 2D correlated spectroscopy (COSY) and 1D NWS spectra are shown in Table 1. A representative 2D COSY spectrum with labeled metabolite and lipid diagonal and cross peaks along with the corresponding ADC map is shown in Figure 1.

### 2.4. Feature Selection

Since there were multiple voxels from the same dataset, some of the statistics were shared between them, especially the adjacent voxels. To avoid data leakage due to the assignments of adjacent voxels to both the training set and testing set, the entire dataset was stratified split into the training and testing sets based on the list of subjects, rather than the voxels. This ensured that the subjects from which the voxels in the training set were derived did not overlap with those of the testing set, and the class distributions were roughly the same in both sets. With a roughly 80–20% train-test split ratio, the training set contained 350 voxels (161 benign and 189 malignant) from 32 datasets, while the test sets contained 86 voxels (14 benign and 18 malignant) from the remaining 8 datasets (3 benign and 5 malignant). This ensured that the samples in the training and testing set were independent, which in turn avoided overestimation of model performance due to data leakage so that the model will be generalizable to new data.

One of the main considerations for the feature selection method was the handling of high-dimensional data with a relatively limited sample size. A statistical significance test was used to narrow down the variable space before running the feature selection algorithms. Only the significant features which were capable of distinguishing benign and malignant classes were selected. Normality and homogeneity of variance of the features were checked using Quantile–Quantile (Q–Q) plots of the data and Levene’s tests. Based on that, either a *t*-test or Mann–Whitney U (MWU) test was used for a statistical significance of *p*-value < 0.01. For the next level of analysis, we considered some of the machine learning model-based feature selection algorithms like sequential feature selection (SFE) and recursive feature elimination (RFE) [54]. SFS and RFE with cross-validation were selected based on the model performance considering all the significant features identified in the statistical test. However, both RFE and SFS have a drawback in that they do not exclude redundant features. This was addressed using a correlation analysis to remove moderate to strong correlated redundant features based on a Spearman’s rank correlation coefficient threshold of ±0.6. A correlation *p*-value < 0.05 was used to check for the statistical significance of the observed correlation between different features. Redundant features with statistically significant high correlation were removed from the feature list.

### 2.5. Machine Learning Algorithms

The open-source machine learning library for Python, ‘scikit-learn’ was used for implementing different supervised learning algorithms for classification [55], which included support vector machine (SVM), Decision Tree, Logistic Regression, Naive Bayes, and K-nearest neighbors (KNN) as well as ensemble learning techniques including Adaptive Boosting (AdaBoost), GradientBoost, Extreme Gradient Boost (XGBoost), Light Gradient Boost, Categorical Boost (CatBoost), RandomForest, and Decision Tree-based bagging classifiers [56,57,58]. In bagging, the training data was divided into different subsets by random sampling with replacement and multiple models were trained on these different subsets. It then combined the prediction of each of the models by averaging. Boosting, on the other hand, used multiple base learners like decision trees in a sequential manner where the successive learner corrected for the error in prediction by the previous one.

### 2.6. Cross-Validation and Parameter Tuning

Grouped K-Folds cross-validation method was used in both feature selection and hyperparameter tuning to return stratified folds with non-overlapping groups that are representative of the class distributions of the dataset. The entire dataset was divided into five non-overlapping folds based on datasets using the Stratified Group 5-Fold method (implemented with the StratifiedGroupKFold method) during the 5-Fold Cross-Validation stage. In each iteration, one of the five folds (20% of the data) was held out to be the testing set, and the remaining folds served as the training set. The cross-validated score was then the average accuracy score across the five folds. The train set was z-score standardized and the test set was standardized with the train set’s statistics. The models were optimized using the cross-validated Grid Search method. Grid search was used by first defining the possible values of hyperparameters in the ML models, and then finding the combination of these parameters that optimize the classification accuracy by exhaustive search.

### 2.7. Evaluation Metrics

The classification performance of the different machine learning models in the testing stage was compared based on the scores of (a) accuracy (ratio of correct predictions to total number of predictions), (b) area under the receiver operating characteristic (ROC) curve (c) precision (True positives/(True positives + False positives)), (d) sensitivity (True positives/(True positives + False negatives)), (e) specificity (True negatives/(True negatives + False positives)) and (f) F1 score (2 × ((precision × sensitivity)/(precision + sensitivity))).

### 2.8. Statistical Analysis

Statistical tests were performed to compare the performance of the machine learning models. One-way Analysis of Variance (ANOVA) test (in RStudio (version 4.1.1)) was used for this comparison, based on the evaluation metrics for a statistical significance level of *p*-value < 0.05. Tukey’s HSD (honestly significant difference) post hoc test was used for pair-wise analysis of these models.

### 2.9. Feature Importance and Model Comparison

Average feature importance was determined by repeating the cross-validation 100 times using the best-performing ensemble models. Then, one-feature models were trained using each of the top features separately to compare the relative classification capability of the individual features. A linear combination of the one-feature models using linear SVM and logistic regression was also studied to show the relative advantage of more complex ML models, like the ensemble models. Five-fold cross-validation was repeated 20 times and the scores were averaged from these 100 repetitions.

## 3. Results

### 3.1. Feature Selection and Comparison

**Figure 2:**
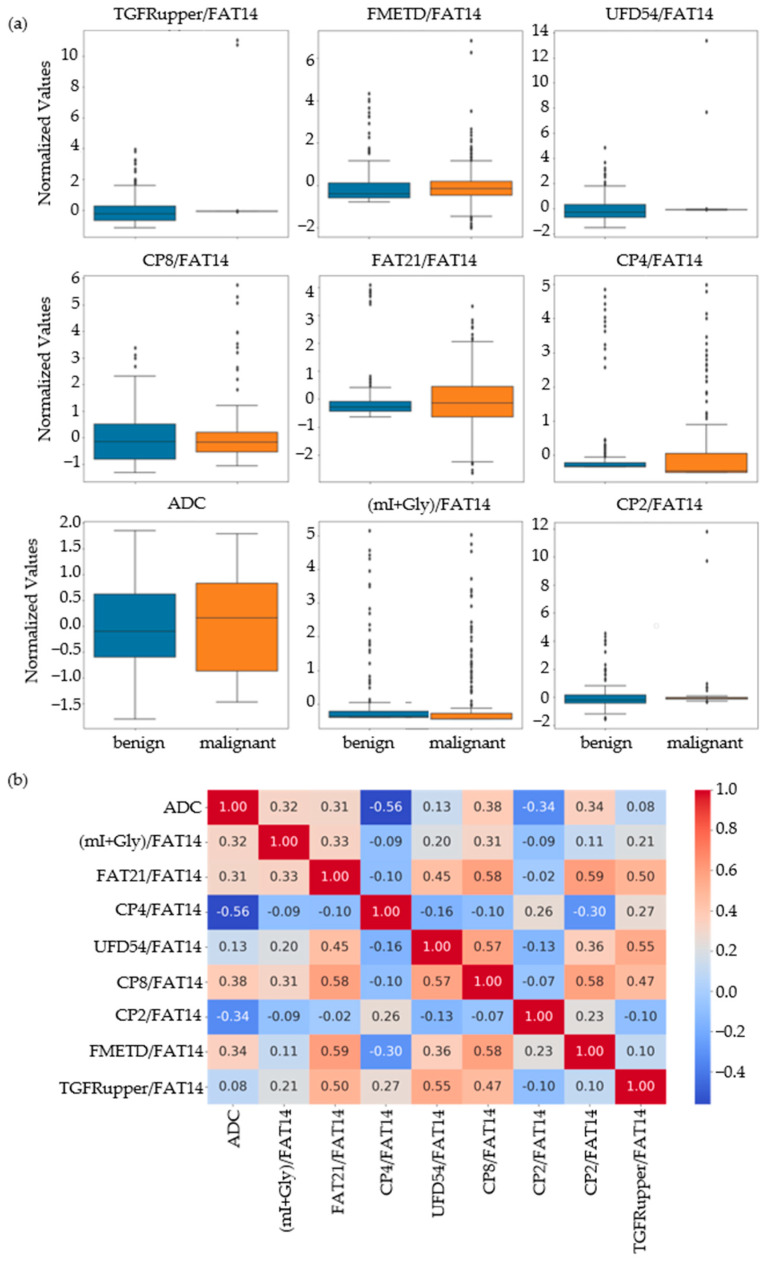
(**a**) Box plots of the most important normalized features selected by correlation analysis, sequential forward selection, and recursive feature elimination processes. (**b**) Spearman’s rank correlation heatmaps of the final set of features.

Based on the results of the MWU test comparing the benign and malignant classes, the feature set was narrowed down to 86 that were statistically significant at *p*-value ≤ 0.01. Nine out of these eighty-six features were identified as the most important by RFE, SFS and correlation analysis. These included ADC and ratios of CP8, FAT21, CP2, FMETD, mI + Gly, CP4, TGFRupper and UFD54 with respect to the diagonal FAT14 peak. The boxplots of these most significant features are shown in Figure 2a for both malignant and benign classes. The larger interquartile range (IQR) of ADC and CP8/FAT14 indicated a larger spread of these features. TGFRupper/FAT14 and UFD54/FAT14, on the other hand, showed the least variability for the malignant class, while CP4/FAT14 showed the least variability for the benign class. The values were z-score normalized. Figure 2b shows the correlation heatmap of these features. Since the feature selection process also included correlation analysis-based exclusion of redundant features, the heatmap showed a correlation coefficient less than 0.6 and greater than −0.06 between any pair of features.

### 3.2. Comparison of Models

**Figure 3:**
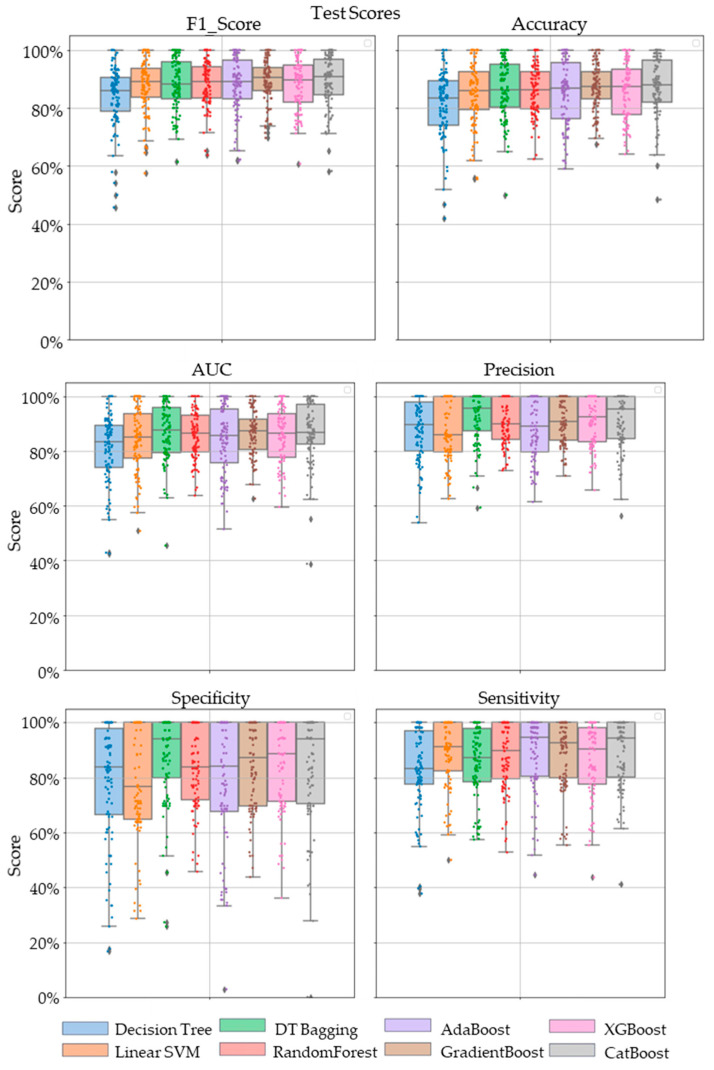
The swarm plots of accuracy, F1 Score, AUC, precision, specificity and sensitivity metrics of the ensemble model during the testing stage. Markers in diamond shape show outliers.

**Figure 4:**
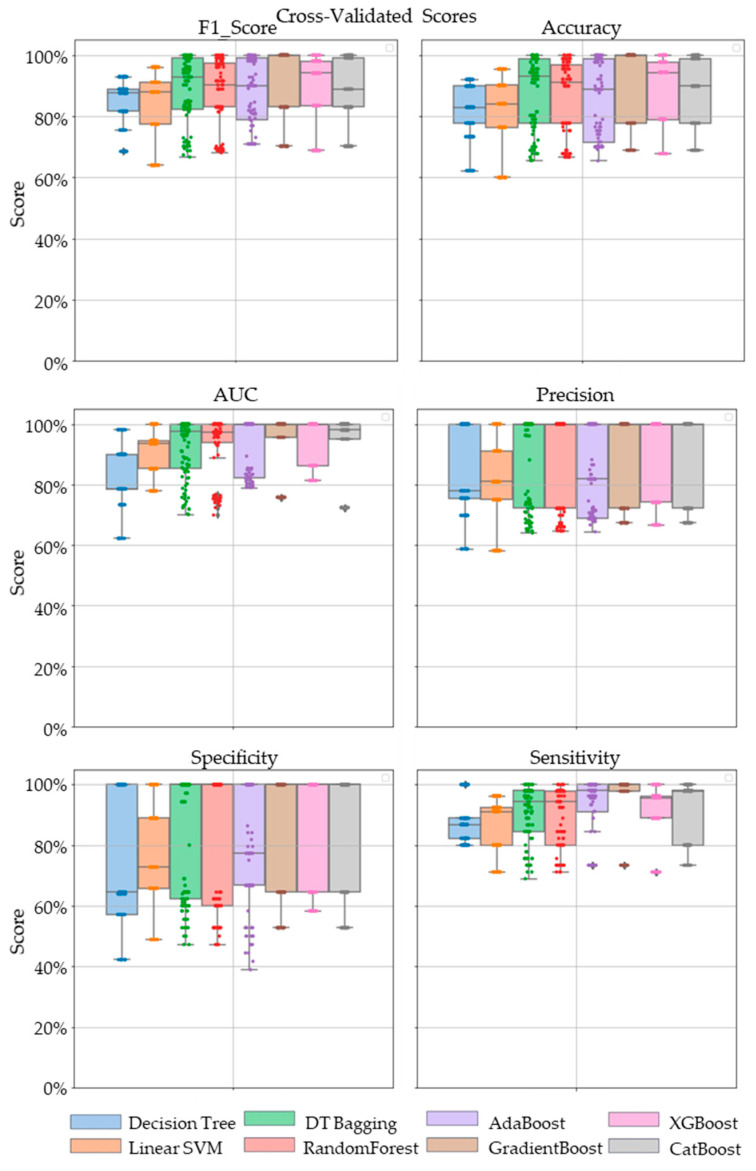
The swarm plots of accuracy, F1 Score, AUC, precision, specificity and sensitivity metrics of the ensemble model during the cross-validation stage. Markers in diamond shape show outliers.

**Figure 5:**
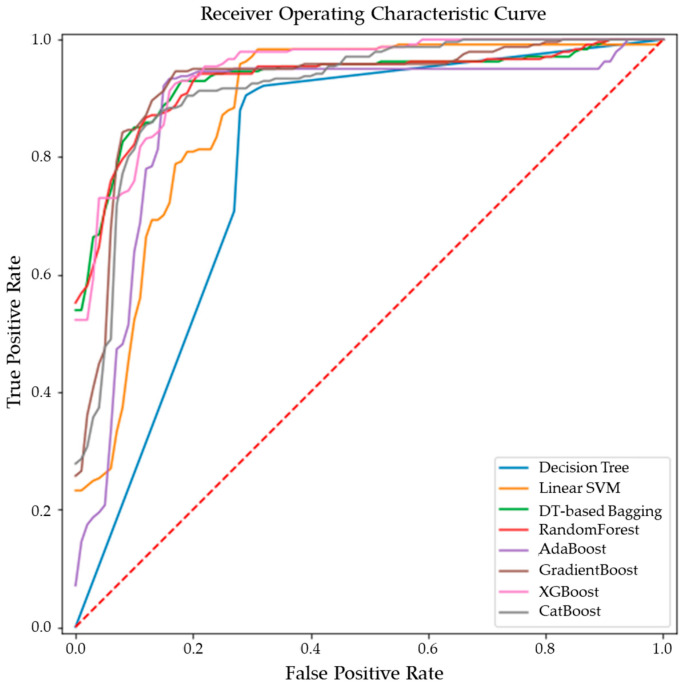
ROC curves of the ensemble models for differentiating malignant from benign breast tissues.

Comparative performance of linear SVM, Decision Tree, DT-based bagging classifier, RandomForest, AdaBoost, GradientBoost, XGBoost and CatBoost are shown in Figure 3, Figure 4 and Figure 5. These models were the best performing out of all the models considered in terms of their accuracy scores. Figure 3 shows the AUC, F1 score, accuracy, precision, sensitivity and specificity of these eight classifiers in the testing stage repeated 100 times with randomized dataset split and model initializations, and Figure 4 shows these scores in the cross-validation stage repeated 50 times using the entire dataset. The respective box plots show the median and IQR of these metrics, along with outliers. Their corresponding mean and standard deviation are listed in Table 2 and the ROC curves of these different models are shown in Figure 5.

While GradientBoost was the model with the highest AUC, accuracy, sensitivity and F1 scores, XGBoost had the maximum precision and specificity as shown in Table 2. However, the results of Tukey’s HSD post hoc test following the ANOVA with *p*-values adjusted for multiple comparisons showed that the differences between the ensemble models XGboost, GradientBoost, CatBoost AdaBoost, Decision Tree based bagging and RandomForest were not statistically significant in terms of Accuracy, AUC, Precision, Sensitivity, Specificity and F1 scores for the significance at *p*-value ≤ 0.05. However, significant differences were observed between the ensemble models and base models like linear SVM and Decision Tree. The ROC curve in Figure 5 also shows a better performance for the ensemble models as compared to the base models, linear SVM and Decision Tree, which is also consistent with the cross-validation scores of other performance metrics shown in Figure 4.

### 3.3. Feature Importance and Linear Combination Models

**Figure 6:**
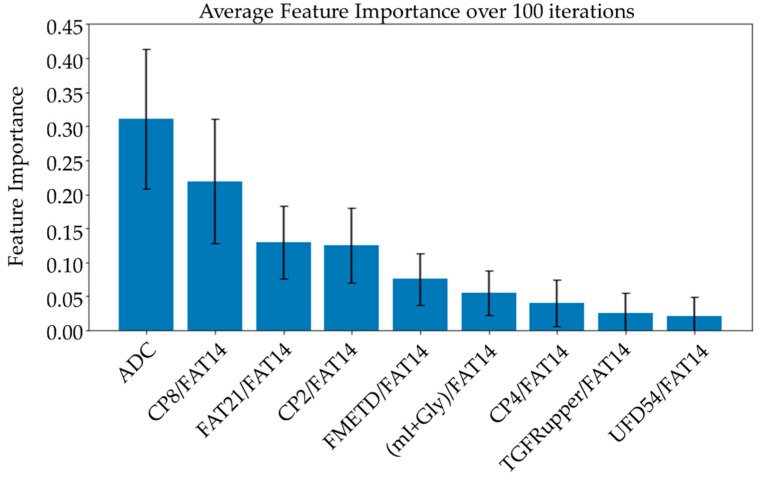
Bar charts showing the average feature importance calculated based on a training set from a randomized train-test split over 100 iterations. Features are arranged in decreasing order of importance from left to right. The error bars indicate standard deviation.

**Figure 7:**
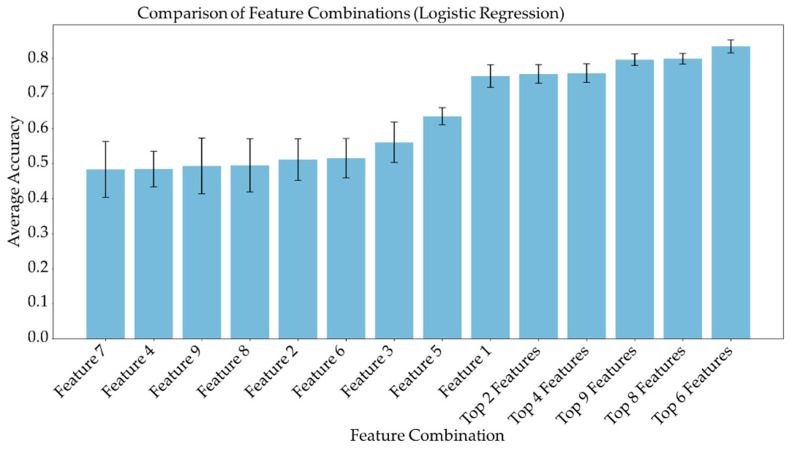
Bar chart showing the average cross-validation accuracy of logistic regression repeated over 100 iterations. Horizontal axis shows different feature combinations used. Features 1 to 9 are ADC and ratios of CP8, FAT21, CP2, FMETD, mI + Gly, CP4, TGFRupper and UFD54 with respect to diagonal FAT14 peak. The error bars indicate standard deviation.

**Figure 8:**
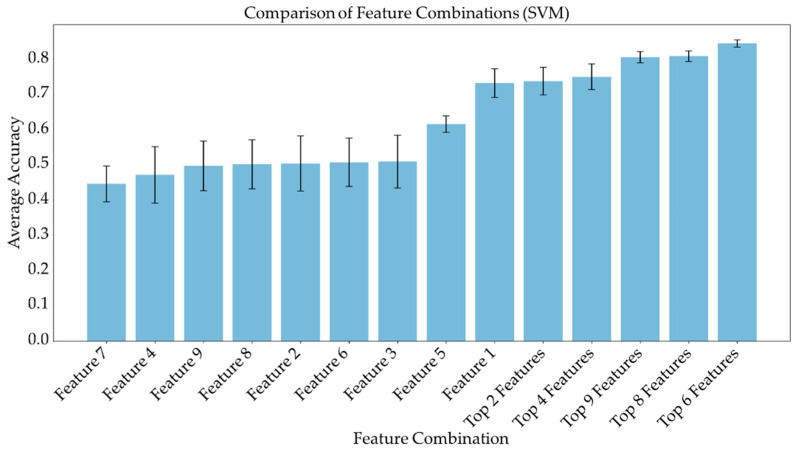
Bar chart showing the average cross-validation accuracy of linear SVM repeated over 100 iterations. Horizontal axis shows different feature combinations used. Features 1 to 9 are ADC and ratios of CP8, FAT21, CP2, FMETD, mI + Gly, CP4, TGFRupper and UFD54 with respect to diagonal FAT14 peak. The error bars indicate standard deviation.

Average feature importance, measured by repeating the cross-validation 100 times using the ensemble models, is shown in Figure 6. Single-feature models were trained and a linear combination of these one-feature classifiers was performed using SVM and logistic regression. Bar charts in Figure 7 and Figure 8 show the average cross-validation accuracy of logistic regression and linear SVM, repeated over 100 iterations. Horizontal axis shows different feature combinations used. Features 1 to 9 are ADC and ratios of CP8, FAT21, CP2, FMETD, mI + Gly, CP4, TGFRupper and UFD54 with respect to the diagonal FAT14 peak. Error bars represent standard deviation. The vertical axis indicates the average accuracy score. The average accuracy of these linear models was reduced when more than the top six features were used, indicating overfitting.

## 4. Discussion

This study showed the feasibility of using metabolite ratios from 5D EP-COSI and ADC values from the DWI data of breast cancer patients to train machine learning models for classifying benign and malignant lesions. While earlier studies have attempted lesion characterization using features extracted from the DWI and DCE-MRI data, these models did not use the quantitative measures of metabolite and lipid features which can be obtained with an MRSI examination [48,49,50]. Although variations in water and fat levels can become ambiguous in glandular regions, especially in benign and healthy tissues, various lipid and metabolite ratios are reported to have statistically significant differences between benign and malignant lesions [42]. Building on this fact, our study pursued a detailed analysis of lesion characterization using 5D EP-COSI features in a machine-learning framework.

The ensemble models were found to perform better than the individual models. This is expected since they combine the strengths of multiple individual models [45]. In fact, the ensemble models can use multiple base models to learn different aspects of the data and hence learn more complex relationships between the variables. They are also more robust to outliers and are also expected to reduce overfitting since they can compensate for the prediction errors of individual models. XGBoost, GradientBoost, RandomForest, AdaBoost and CatBoost were found to be the best-performing ensemble models in this study with 92% to 95% AUC, 86% to 90% accuracy, 87% to 89% F1 scores, 84% to 89% precision, 89% to 95% sensitivity and 79% to 85% specificity. While the highest sensitivity of 94.2% was achieved with GradientBoost, the highest specificity of 84.6% was achieved using XGBoost, which is higher than the reported performance metrics of DWI or MRSI without the application of machine learning techniques [37,38,39,40].

While the feature importance scores slightly varied among the top-performing models, ADC was ranked first on average over 100 iterations using different ensemble models. Four out of the top nine features were the ratios of cross-peaks, which are specific to the 2D COSY technique. The remaining four main features were the ratios of diagonal lipid peaks. It is interesting to note that the ratios of lipid cross peaks ranked higher than some of the conventional biomarkers like Cho and mI + Gly ratios for classifying benign and malignant lesions in the ML framework. While both Cho and mI + Gly ratios were in the list of statistically significant variables in the MWU tests, only mI + Gly ratio was selected in the top nine features. This is mainly due to the high correlation between the two features. Therefore, Cho may also be used in place of mI + Gly, or a combination of the two could be used as a single feature to achieve a similar classification performance. The same argument exists for some of the lipid peaks as well, for example, different fat peaks in the range of 1 to 2 ppm could be highly correlated, especially with large linewidths and quantitation by peak integration.

ADC and CP8/FAT14 had the highest average feature importance scores. Linear SVM and logistic regression favored ADC more than other features. This could be because the linear classification models like linear SVM and logistic regression favored a more linear relationship between ADC and the target classes. Linear combination of the one-feature models showed a maximum average accuracy less than that of ensemble models and was found to be dropping with the increased number of features. Possible reasons include the complex relationship of the features and reduced ratio of data points to features. Other possible factors like redundancy and relevancy of the individual features are less likely to be the cause since the correlated features were not included and these features were known to be relevant to breast cancer-related changes in cellular metabolism and tissue structure. This indicates that a better classification would require advanced models capable of learning non-linear relationships like the ensemble machine learning models studied in this work and can also benefit from more data points.

The number of datasets is one of the limitations of this study. Even though we have multiple voxels from the same dataset giving metabolite and lipid ratios, it is important to split the data based on the actual number of subjects rather than the voxels. It would be tempting to consider the individual voxels as separate data when splitting the data into training and testing sets. However, this approach could lead to severe data leakage, since multiple voxels from the same subject can have similar statistics, especially when interpolation is used to increase the number of voxels. Otherwise, if the lesion spans multiple voxels in the spectroscopic data, the relatively low resolution and partial volume effects can potentially cause slightly overlapping information between the neighboring voxels. Therefore, if the train-test split is performed based on the voxels rather than individual subjects, it is reasonable to assume that during the training stage, the model would already see some of the statistics present in the testing data. This will artificially increase the score of test and validation performance metrics but will not be generalizable to a new subject.

Even though these ML models should be generalizable to the MRSI/DWI data from different scanners and sites, it may be considered as another limitation of this study since there could be subtle/complex variations in the datasets from different scanners and sites so that the list of most important features could differ. A future study with a larger sample size, ideally from different scanners and sites, can further validate the results presented in this work.

Since the focus of this study was to analyze the performance of ML models with features from the 5D EP-COSI data, we have not considered some of the image-based features potentially available from DWI. For example, it has been recently shown that the features based on continuous-time random-walk (CTRW) and intravoxel incoherent motion (IVIM) models from DWI using multiple b-values can classify benign and malignant breast lesions using ensemble ML models [48]. More radiomics features from DWI as well as other modalities like DCE-MRI can be used in a future study to potentially further improve the model performance.

## 5. Conclusions

In this pilot validation of the multi-dimensional (5D EP-COSI) data for the characterization of breast tissues, we have shown that ML-based classification models can be trained using spectroscopic features in conjunction with ADC values from DWI to classify benign and malignant lesions. Multiple diagonal and cross-peaks from 2D COSY spectra were identified as important features, further asserting the advantage of 2D COSY spectra as compared to features derived from 1D spectra. GradientBoost, CatBoost, RandomForest AdaBoost and XGBoost were the best performing models with 92% to 95% AUC, 86% to 90% accuracy, 87% to 89% F1 scores, 84% to 89% precision, 89% to 95% sensitivity and 79% to 85% specificity.

## Figures and Tables

**Table 1:** Metabolites and lipids identified in the 2D COSY and 1D NWS spectra of breast tissue.

2D COSY				1D NWS	
Diagonal Peaks		Cross-Peaks		Peak Label	Locations (F_2_) ppm
Peak Label	Locations (F_2_, F_1_) ppm	Peak Label	Locations (F_2_, F_1_) ppm		
Methyl Fat (FMETD)	(0.9, 0.9)	CP1	(0.9, 1.4)	Methylene Fat (FAT14_1d)	1.4
Methylene Fat (FAT14)	(1.4, 1.4)	CP2	(1.4, 0.9)	Water (WAT_1d)	4.7
Methylene Fat (FAT21)	(2.1, 2.1)	CP3	(1.6, 2.3)	Olefinic Fat (UFD54_1d)	5.4
Methylene Fat (FAT23)	(2.3, 2.3)	CP4	(2.3, 1.6)		
Methylene Fat (FAT29)	(2.9, 2.9)	CP5	(1.4, 2.1)		
Choline (Cho)	(3.2, 3.2)	CP6	(2.1, 1.4)		
myo-Inositol + Glycine (mI + Gly)	(3.5, 3.5)	CP7	(4.1, 4.3)		
Methylene Glycerol Backbone (MGB41)	(4.1, 4.1)	CP8	(4.3, 4.1)		
Methylene Glycerol Backbone (MGB43)	(4.3, 4.3)	Unsaturated fatty acid cross peak, right lower (UFR_lower)	(2.1, 5.4)		
Water (WAT)	(4.7, 4.7)	Unsaturated fatty acid cross peak, left lower (UFL_lower)	(2.9, 5.4)		
Olefinic Fat (UFD54)	(5.4, 5.4)	Triglyceryl fat cross peak lower, (TGF_lower)	(4.2, 5.3)		
		Unsaturated fatty acid cross peak, right upper (UFR_upper)	(5.4, 2.1)		
		Unsaturated fatty acid cross peak, left upper (UFL_upper)	(5.4, 2.9)		
		Triglyceryl fat cross peak upper (TGF_upper)	(5.3, 4.2)		

**Table 2:** Mean and standard deviation of AUC, accuracy, F1 Score, precision, specificity and sensitivity scores of different machine learning models. Highest value is each column is shown in bold.

Model	AUC (%)	Accuracy (%)	F1 score (%)	Precision (%)	Sensitivity (%)	Specificity (%)
AdaBoost	92.77 ± 9.02	86.43 ± 12.43	88.10 ± 11.20	84.48 ± 13.96	92.79 ± 10.22	79.17 ± 19.93
CatBoost	93.09 ± 10.56	87.08 ± 12.15	88.23 ± 11.08	87.90 ± 14.98	89.84 ± 11.05	83.44 ± 20.71
DT-based Bagging	92.20 ± 9.67	87.30 ± 11.95	88.63 ± 10.86	87.74 ± 14.72	90.49 ± 9.55	83.35 ± 19.94
Decision Tree	82.82 ± 10.50	82.31 ± 8.80	84.75 ± 7.23	83.76 ± 14.19	87.58 ± 7.00	75.32 ± 21.03
GradientBoost	**94.28 ± ** **9.44**	**89.33 ± ** **13.43**	**90.65 ± ** **12.21**	87.90 ± 14.98	**94.22 ± ** **10.53**	83.44 ± 20.71
Linear SVM	90.24 ± 7.81	81.21 ± 12.42	83.31 ± 11.48	81.05 ± 14.35	86.18 ± 9.32	75.24 ± 17.93
RandomForest	93.40 ± 9.59	86.39 ± 12.17	87.78 ± 11.01	87.51 ± 15.50	89.31 ± 10.08	82.76 ± 21.50
XGBoost	93.50 ± 8.11	87.78 ± 12.46	88.90 ± 11.61	**88.16 ± ** **14.77**	90.36 ± 10.32	**84.56 ± ** **19.11**

## Data Availability

The datasets generated and/or analyzed during the current study are not publicly available due to ethical and data protection restrictions, but are available from corresponding author on reasonable request and subject to an institutional data sharing agreement.

## References

[1] Siegel R.L., Miller K.D., Wagle N.S., Jemal A. (2023). Cancer statistics, 2023. CA Cancer J. Clin..

[2] Bray F., Ferlay J., Soerjomataram I., Siegel R., Torre L., Jemal A. (2020). Erratum: Global cancer statistics 2018: GLOBOCAN estimates of incidence and mortality worldwide for 36 cancers in 185 countries. CA Cancer J. Clin..

[3] Al-Ajmi K., Lophatananon A., Yuille M., Ollier W., Muir K.R. (2018). Review of non-clinical risk models to aid prevention of breast cancer. Cancer Causes Control.

[4] Fu B., Liu P., Lin J., Deng L., Hu K., Zheng H. (2018). Predicting invasive disease-free survival for early stage breast cancer patients using follow-up clinical data. IEEE Trans. Biomed. Eng..

[5] Jagannathan N. (2009). Breast MR. NMR Biomed..

[6] Lehman C.D., Isaacs C., Schnall M.D., Pisano E.D., Ascher S.M., Weatherall P.T., Bluemke D.A., Bowen D.J., Marcom P.K., Armstrong D.K. (2007). Cancer yield of mammography, MR, and US in high-risk women: Prospective multi-institution breast cancer screening study. Radiology.

[7] Morris E.A. (2007). Diagnostic breast MR imaging: Current status and future directions. Radiol. Clin. N. Am..

[8] Pe M., Dorme L., Coens C., Basch E., Calvert M., Campbell A., Cleeland C., Cocks K., Collette L., Dirven L. (2018). Statistical analysis of patient-reported outcome data in randomised controlled trials of locally advanced and metastatic breast cancer: A systematic review. Lancet Oncol..

[9] Pop C.F., Stanciu-Pop C., Drisis S., Radermeker M., Vandemerckt C., Noterman D., Moreau M., Larsimont D., Nogaret J.M., Veys I. (2018). The impact of breast MRI workup on tumor size assessment and surgical planning in patients with early breast cancer. Breast J..

[10] Saslow D., Boetes C., Burke W., Harms S., Leach M.O., Lehman C.D., Morris E., Pisano E., Schnall M., Sener S. (2007). American Cancer Society guidelines for breast screening with MRI as an adjunct to mammography. CA Cancer J. Clin..

[11] Weinreb J.C., Newstead G. (1995). MR imaging of the breast. Radiology.

[12] Zhang M., Horvat J.V., Bernard-Davila B., Marino M.A., Leithner D., Ochoa-Albiztegui R.E., Helbich T.H., Morris E.A., Thakur S., Pinker K. (2019). Multiparametric MRI model with dynamic contrast-enhanced and diffusion-weighted imaging enables breast cancer diagnosis with high accuracy. J. Magn. Reson. Imaging.

[13] Warner E. (2011). Breast-cancer screening. N. Engl. J. Med..

[14] Aboagye E.O., Bhujwalla Z.M. (1999). Malignant transformation alters membrane choline phospholipid metabolism of human mammary epithelial cells. Cancer Res..

[15] Bolan P.J., Kim E., Herman B.A., Newstead G.M., Rosen M.A., Schnall M.D., Pisano E.D., Weatherall P.T., Morris E.A., Lehman C.D. (2017). MR spectroscopy of breast cancer for assessing early treatment response: Results from the ACRIN 6657 MRS trial. J. Magn. Reson. Imaging.

[16] Dorrius M.D., Pijnappel R.M., Jansen-van der Weide M.C., Jansen L., Kappert P., Oudkerk M., Sijens P.E. (2011). Determination of choline concentration in breast lesions: Quantitative multivoxel proton MR spectroscopy as a promising noninvasive assessment tool to exclude benign lesions. Radiology.

[17] Gribbestad I., Sitter B., Lundgren S., Krane J., Axelson D. (1999). Metabolite composition in breast tumors examined by proton nuclear magnetic resonance spectroscopy. Anticancer Res..

[18] Haukaas T.H., Euceda L.R., Giskeødegård G.F., Bathen T.F. (2017). Metabolic portraits of breast cancer by HR MAS MR spectroscopy of intact tissue samples. Metabolites.

[19] Jagannathan N., Seenu V., Kumar M. (2002). Potential of in vivo proton MR spectroscopy in the assessment of breast lesions without the use of contrast agent. Radiology.

[20] Roebuck J.R., Cecil K.M., Schnall M.D., Lenkinski R.E. (1998). Human breast lesions: Characterization with proton MR spectroscopy. Radiology.

[21] Sharma U., Mehta A., Seenu V., Jagannathan N. (2004). Biochemical characterization of metastatic lymph nodes of breast cancer patients by in vitro 1H magnetic resonance spectroscopy: A pilot study. Magn. Reson. Imaging.

[22] Thakur S.B., Horvat J.V., Hancu I., Sutton O.M., Bernard-Davila B., Weber M., Oh J.H., Marino M.A., Avendano D., Leithner D. (2019). Quantitative in vivo proton MR spectroscopic assessment of lipid metabolism: Value for breast cancer diagnosis and prognosis. J. Magn. Reson. Imaging.

[23] Thomas M.A., Binesh N., Yue K., DeBruhl N. (2001). Volume-localized two-dimensional correlated magnetic resonance spectroscopy of human breast cancer. J. Magn. Reson. Imaging.

[24] Amornsiripanitch N., Nguyen V.T., Rahbar H., Hippe D.S., Gadi V.K., Rendi M.H., Partridge S.C. (2018). Diffusion-weighted MRI characteristics associated with prognostic pathological factors and recurrence risk in invasive ER+/HER2–breast cancers. J. Magn. Reson. Imaging.

[25] Bammer R. (2003). Basic principles of diffusion-weighted imaging. Eur. J. Radiol..

[26] Belli P., Costantini M., Bufi E., Magistrelli A., La Torre G., Bonomo L. (2010). Diffusion-weighted imaging in breast lesion evaluation. Radiol. Med..

[27] Delbany M., Bustin A., Poujol J., Thomassin-Naggara I., Felblinger J., Vuissoz P.A., Odille F. (2019). One-millimeter isotropic breast diffusion-weighted imaging: Evaluation of a superresolution strategy in terms of signal-to-noise ratio, sharpness and apparent diffusion coefficient. Magn. Reson. Med..

[28] deSouza N.M. (2018). Diffusion-weighted MRI in multicenter trials of breast cancer: A useful measure of tumor response?. Radiol. Soc. N. Am..

[29] Le Bihan D., Breton E., Lallemand D., Grenier P., Cabanis E., Laval-Jeantet M. (1986). MR imaging of intravoxel incoherent motions: Application to diffusion and perfusion in neurologic disorders. Radiology.

[30] Newitt D.C., Zhang Z., Gibbs J.E., Partridge S.C., Chenevert T.L., Rosen M.A., Bolan P.J., Marques H.S., Aliu S., Li W. (2019). Test–retest repeatability and reproducibility of ADC measures by breast DWI: Results from the ACRIN 6698 trial. J. Magn. Reson. Imaging.

[31] Sharma U., Danishad K.K.A., Seenu V., Jagannathan N.R. (2009). Longitudinal study of the assessment by MRI and diffusion-weighted imaging of tumor response in patients with locally advanced breast cancer undergoing neoadjuvant chemotherapy. NMR Biomed. Int. J. Devoted Dev. Appl. Magn. Reson. In Vivo.

[32] Furman-Haran E., Grobgeld D., Kelcz F., Degani H. (2001). Critical role of spatial resolution in dynamic contrast-enhanced breast MRI. J. Magn. Reson. Imaging.

[33] Hickman P., Moore N., Shepstone B. (1994). The indeterminate breast mass: Assessment using contrast enhanced magnetic resonance imaging. Br. J. Radiol..

[34] Kvistad K.A., Rydland J., Vainio J., Smethurst H.B., Lundgren S., Fjøsne H.E., Haraldseth O. (2000). Breast lesions: Evaluation with dynamic contrast-enhanced T1-weighted MR imaging and with T2*-weighted first-pass perfusion MR imaging. Radiology.

[35] Liu P., Debatin J., Caduff R., Kacl G., Garzoli E., Krestin G. (1998). Improved diagnostic accuracy in dynamic contrast enhanced MRI of the breast by combined quantitative and qualitative analysis. Br. J. Radiol..

[36] Millet I., Pages E., Hoa D., Merigeaud S., Curros Doyon F., Prat X., Taourel P. (2012). Pearls and pitfalls in breast MRI. Br. J. Radiol..

[37] Bogner W., Gruber S., Pinker K., Grabner G., Stadlbauer A., Weber M., Moser E., Helbich T.H., Trattnig S. (2009). Diffusion-weighted MR for differentiation of breast lesions at 3.0 T: How does selection of diffusion protocols affect diagnosis?. Radiology.

[38] Chen X., Li W.-L., Zhang Y.-L., Wu Q., Guo Y.-M., Bai Z.-L. (2010). Meta-analysis of quantitative diffusion-weighted MR imaging in the differential diagnosis of breast lesions. BMC Cancer.

[39] Prvulovic Bunovic N., Sveljo O., Kozic D., Boban J. (2021). Is Elevated Choline on Magnetic Resonance Spectroscopy a Reliable Marker of Breast Lesion Malignancy?. Front. Oncol..

[40] Shahraki Z., Ghaffari M., Parooie F., Salarzaei M. (2022). Preoperative evaluation of breast cancer: Contrast-enhanced mammography versus contrast-enhanced magnetic resonance imaging: A systematic review and meta-analysis. Breast Dis..

[41] Baltzer P.A., Dietzel M. (2013). Breast lesions: Diagnosis by using proton MR spectroscopy at 1.5 and 3.0 T—Systematic review and meta-analysis. Radiology.

[42] Joy A., Saucedo A., Joines M., Lee-Felker S., Kumar S., Sarma M.K., Sayre J., DiNome M., Thomas M.A. (2022). Correlated MR spectroscopic imaging of breast cancer to investigate metabolites and lipids: Acceleration and compressed sensing reconstruction. BJR|Open.

[43] Aamir S., Rahim A., Aamir Z., Abbasi S.F., Khan M.S., Alhaisoni M., Khan M.A., Khan K., Ahmad J. (2022). Predicting breast cancer leveraging supervised machine learning techniques. Comput. Math. Methods Med..

[44] Dou Y., Meng W. (2021). An Optimization Algorithm for Computer-Aided Diagnosis of Breast Cancer Based on Support Vector Machine. Front. Bioeng. Biotechnol..

[45] Greener J.G., Kandathil S.M., Moffat L., Jones D.T. (2022). A guide to machine learning for biologists. Nat. Rev. Mol. Cell Biol..

[46] Zhou Z.-H., Zhou Z.-H. (2021). Ensemble Learning.

[47] Qi C., Li Y., Fan X., Jiang Y., Wang R., Yang S., Meng L., Jiang T., Li S. (2019). A quantitative SVM approach potentially improves the accuracy of magnetic resonance spectroscopy in the preoperative evaluation of the grades of diffuse gliomas. NeuroImage Clin..

[48] Mehta R., Bu Y., Zhong Z., Dan G., Zhong P.-S., Zhou C., Hu W., Zhou X.J., Xu M., Wang S. (2023). Characterization of breast lesions using multi-parametric diffusion MRI and machine learning. Phys. Med. Biol..

[49] Parekh V.S., Jacobs M.A. (2017). Integrated radiomic framework for breast cancer and tumor biology using advanced machine learning and multiparametric MRI. NPJ Breast Cancer.

[50] Daimiel Naranjo I., Gibbs P., Reiner J.S., Lo Gullo R., Sooknanan C., Thakur S.B., Jochelson M.S., Sevilimedu V., Morris E.A., Baltzer P.A. (2021). Radiomics and machine learning with multiparametric breast MRI for improved diagnostic accuracy in breast cancer diagnosis. Diagnostics.

[51] Klose U. (1990). In vivo proton spectroscopy in presence of eddy currents. Magn. Reson. Med..

[52] Burns B.L., Wilson N.E., Thomas M.A. (2014). Group sparse reconstruction of multi-dimensional spectroscopic imaging in human brain in vivo. Algorithms.

[53] Wilson N.E., Burns B.L., Iqbal Z., Thomas M.A. (2015). Correlated spectroscopic imaging of calf muscle in three spatial dimensions using group sparse reconstruction of undersampled single and multichannel data. Magn. Reson. Med..

[54] Guyon I., Weston J., Barnhill S., Vapnik V. (2002). Gene selection for cancer classification using support vector machines. Mach. Learn..

[55] Pedregosa F., Varoquaux G., Gramfort A., Michel V., Thirion B., Grisel O., Blondel M., Prettenhofer P., Weiss R., Dubourg V. (2011). Scikit-learn: Machine learning in Python. J. Mach. Learn. Res..

[56] Buitinck L., Louppe G., Blondel M., Pedregosa F., Mueller A., Grisel O., Niculae V., Prettenhofer P., Gramfort A., Grobler J. (2013). API design for machine learning software: Experiences from the scikit-learn project. arXiv.

[57] Chen T., Guestrin C. Xgboost: A scalable tree boosting system. Proceedings of the 22nd Acm Sigkdd International Conference on Knowledge Discovery and Data Mining.

[58] Dorogush A.V., Ershov V., Gulin A. (2018). CatBoost: Gradient boosting with categorical features support. arXiv.

